# Infant mortality in the Flemish Region of Belgium 1999-2008: a time-to-event analysis

**DOI:** 10.1186/0778-7367-70-6

**Published:** 2012-03-30

**Authors:** Edwin Pelfrene, Heidi Cloots, Erik Hendrickx

**Affiliations:** 1 Studiedienst van de Vlaamse Regering (SVR) [Study Centre of the Government of Flanders], Vlaamse overheid [Flemish authorities], Boudewijnlaan, 30, 1000 Brussels, Belgium; 2 Vlaams Agentschap Zorg en Gezondheid (VAZG) [Flemish Agency for Care & Health], Vlaamse overheid [Flemish authorities], Brussels, Belgium

**Keywords:** Belgium, Flemish Region, Infant mortality, Life expectancy at birth

## Abstract

**Background:**

When calculating life expectancy, it is usually assumed that deaths are uniformly distributed within each of the age intervals. As most of the infant deaths are neonatal deaths, this calls for a better assessment for that age group.

**Methods:**

The Flemish unified death and birth certificates database for all calendar years between 1999 and 2008 was used. A Kaplan-Meier survival analysis on a yearly basis was performed to assess the mean time-to-event and to compare survival curves between both genders.

**Results:**

Over the last years, a slight though not steady decrease of the infant mortality rate is observed. In 2008, the probability among live births of dying before their first anniversary is 4.6‰ in boys and 3.5‰ in girls. The large majority (about 85%) of these have died in their year of birth. The mean survival time of deaths in their year of birth was found to centre around 1 month (about 30 days), which results in a 'mean proportion of the calendar year lived' *(k1) *close to 0.09. Among those who died in the year after their year of birth yet before their first anniversary, no such concentration in time of the deaths is observed. Differences between the gender groups are small and generally not statistically significant.

**Conclusion:**

*Statistics Belgium*, the federal statistics office, imputes a value for k1 equal to 0.1 for infant deaths in their year of birth when calculating life expectancy. Our data fully support this value. We think such refinement is generally feasible in calculating life expectancy.

## Background

### Objective

When calculating life expectancy, it is assumed that deaths are uniformly distributed within each of the age intervals, which translates into the imputation of an additional 0.5 years of life for the deceased in their year of death. This generally holds for all ages, except for the youngest age group, and probably for the oldest age group as well (above 80) [1-3].

Looking at infant mortality, the striking feature is indeed that most of the deaths among live births are concentrated in the very first days. This fact urges us to adopt some factor *k *notably inferior to 0.5 for the mean proportion of the calendar year lived by infants who die in their first year of life.

Our aim is to assess this factor *k *by analyzing data for the Flemish Region in Belgium. Which kinds of *k*-factor(s) should be considered, however, depends on the sort of life table used.

### Location of k-factors within the life table

Usually, life expectancies are derived from so-called period life tables in which age-specific mortality risks based on observations that occurred within successive birth cohorts in a given period of time (typically a calendar year), are applied to one hypothetical birth cohort under the assumption that the risks do not change over time.

Two models of period life tables can be distinguished, depending on the kind of age groups that are observed: a) one with the age at the start of the calendar year (or equivalently, the age 'attained' at the end of the calendar year), and b) one with the age at the last birthday [2,4]. This is also referred to as age expressed in completed years versus age in exact years, respectively.

**Figure 1:**
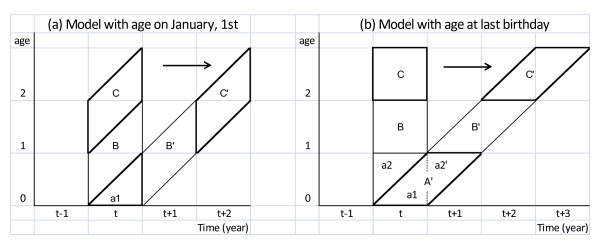
**Lexis diagram**. Lexis diagram for observations in the calendar year *t *and its projection on the hypothetical cohort, in a model (a) with age attained on January 1st and (b) with age at last birthday.

Figure 1 illustrates on a Lexis-diagram, with calendar year on the x-axis and age on the y-axis, the way in which the successive birth cohorts build up the hypothetical birth cohort in both models.

To calculate life expectancy (at birth), it is necessary to ascertain correct values for the person-years lived in each of the discerned parallelograms of the hypothetical cohort in both models, and in the case of the model with age reached on January 1^st^, also in its base triangle a1. In doing so, it is noteworthy that in model (a) with age attained on January 1^st^, each parallelogram depicting one age group or birth cohort actually covers 2 ages, whereas in model (b) with age at last birthday, each age group covers 2 birth cohorts (suitably projected on 2 calendar years in the hypothetical birth cohort).

In model (a), we assume that the newborns of year *t *who survive until the end of the year, will on average have lived 0.5 years insofar as births are uniformly spread over the entire calendar year. This can be deduced from the length of the midline connecting the midpoints of the rectangular sides in triangle a1. On the other hand, the newborns of year *t *who have died in the set time interval depicted by triangle a1, will on average have lived some observed time length equal to *k1 *years, with *k1 *less than 0.5, or even less than the expected value (0.25) for that time interval, given uniform distributions of births and deaths.

In model (b), parallelogram A' shows on the hypothetical cohort that the newborns of year *t *who reach their first anniversary, will all have lived 1 year. The infants who died in their first year of life, will either have died before the end of their year of birth (in triangle a1), or else in the next year before their first anniversary (in triangle a2'). The mean proportion of the calendar year lived by the deceased infants is then the weighted average of the mean proportions observed in both discerned periods, that is *k *= k1*w1 + k2*w2. In this, *k1 *refers to the mean proportion of a calendar year lived by the deceased in the base triangle (a1) and *k2 *refers to the mean proportion of a calendar year lived since birth by the deceased in the next triangle (a2') during their imagined passage through parallelogram A' (comprising both triangles). The weights *w1 *and *w2 *then refer to the proportion of infant deaths in the year of birth or in the next year before the first anniversary, respectively. From the figure, it should be clear however that *k2 *and w2 are actually derived from observations made in the former birth cohort (that is, in triangle a2 within the base square of the observation year).

## Methods

### Database

The data source for this research is the Flemish unified death and birth certificates database, which is operated by the Flemish central administration. This contains data of all live births and all deaths of infants with a legal residence in the Flemish Region, that were registered in either the Flemish or Brussels Capital Regions. It includes births and deaths in the resident refugee population.

More particularly, our analyses include the following data for all years of birth between 1999 and 2008:

• the number of registered live births for mothers with legal residence in the Flemish Region, by year of birth and by gender;

• the number of registered deaths of infants with legal residence in the Flemish Region that died in their year of birth (excluding still births). This is broken down by the number of days lived, by year of birth and by gender;

• the number of registered deaths of infants with legal residence in the Flemish Region that died in the year following their year of birth but before their first anniversary. This is broken down by number of days lived, by year of birth and by gender.

### Survival analysis

To examine the time-to-event of interest, i.e. the number of days lived by the deceased either in their year of birth or in the following year before the first anniversary, a survival analysis was performed using SPSS 16.0 for Windows. More particularly the Kaplan-Meier procedure was applied, which makes it possible to compare survival distributions among subgroups. To test the equality of the survival curves, the Breslow chi-square statistic is reported in which time points are weighted by the number of cases at risk at that time point.

It is important to note that 'the number of days lived' were recorded as 'completed days', i.e. those who died on their birthday have 0 days on their record, those who died the very next day have 1 day on their record, etc. Assuming a uniform distribution of deaths within the calendar day, this (again) leads to the substitution of an average of 0.5 days lived for those who died on their birthday, an average of 1.5 days for those who died the following day, etc. These averages on a daily basis were duly taken into account.

**Figure 2:**
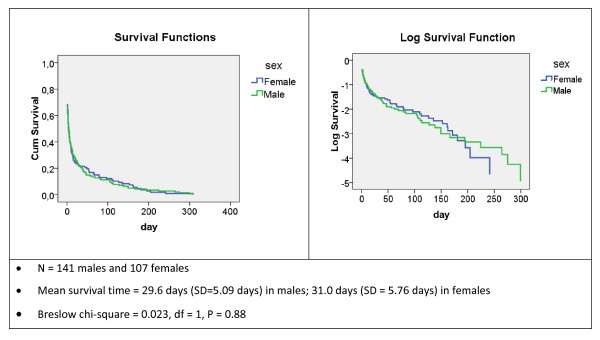
**Survival curves**. Survival curves for the deceased in their year of birth, Flemish Region (Belgium), by sex, 2008.

Graphs of the survival functions within the year of birth are pictured, both on a linear scale and a log scale, the latter being more apt to picture small differences between subgroups (Figure 2).

The mean proportion of the calendar year lived by the deceased in the year of birth (*k1*) was derived from their mean survival time (in days). Likewise, the mean proportion of the calendar year lived by the deceased in the year after the year of birth yet before the first anniversary (*k2*), was derived from their mean survival time since birth (in days).

Differences in proportions were tested with the usual independent samples t-test, assuming the validity of the central limit theorem for large samples. Only the *P*-value is reported. The usual level of significance is adopted (α = 0.05).

## Results

### Infant mortality rates

Between 1999 and 2008, the number of registered live births per annum for mothers having their residence in the Flemish Region roughly ranged between 60,000 and 70,000, with boys slightly outnumbering girls (sex ratio close to 1.05). The lowest number of births was recorded in 2002 (60,161), the highest in 2008 (69,276).

**Figure 3:**
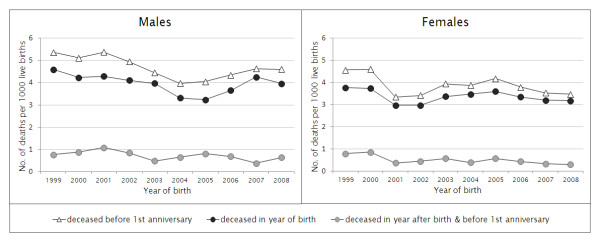
**Probability of infant mortality**. Probability of infant mortality in the Flemish Region (Belgium) according to the year of birth, by sex, 1999-2008.

Figure 3 shows the probability of infant mortality by gender, i.e. the probability among registered live births of dying before the first anniversary. A slight though not steady decrease is observed over the years: from 5.4 in 1999 to 4.6 deaths per thousand live births in 2008 (-14%) for boys, and from 4.6 in 1999 to 3.5 per thousand live births in 2008 (-24%) for girls.

In addition, the figure displays (a) the probability of dying in the year of birth and (b) the probability of dying in the next year before the infant's first anniversary. This clearly shows that the large majority of those who died before their first anniversary actually did so in their year of birth. The average share for all observation years is 85% in males and 87% in females (*P *= 0.68).

The probability of dying in the year of birth decreases from 4.6 to 4.0 per thousand live births (‰) between 1999 and 2008 in boys and from 3.8 to 3.2‰ in girls, with some fluctuations over that period. Note that a significant difference between both gender groups was found only for the years 2001 (*P *= 0.0071) and 2002 (*P *= 0.019).

The probability of dying after the year of birth but before the first anniversary is much lower, with stable mortality rates well below 1 per thousand. The average for all observation years is 0.7‰ in males and 0.5‰ in females (*P *= 0.34).

### Mean time-to-event in the year of birth

Figure 2 displays, for the calendar year 2008, the survival curves by gender in the year of birth according to the days lived. Most of the deaths are concentrated in the first days of life. Half of the infants who were born and died in 2008 lived less than 1 week (the median survival time is 4.5 days in males and 3.5 days in females). The mean survival time for that year is equal to 29.6 days for the deceased boys and 31.0 days for the deceased girls. Expressed as a proportion of the calendar year, this gives *k1 *equal to 0.081 in males and 0.085 in females. Note that the difference in survival graphs between both gender groups is not statistically significant.

Table 1 summarizes the mean survival time value of the deceased in their year of birth as well as the resulting mean proportion lived during that year (*k1*) for all observation years by gender. The value of *k1 *is quite stable over the years, on average 0.085 in males and 0.090 in females. Between the gender groups, no significant differences in survival graphs are reported.

**Table 1:** Mean survival time of the deceased in their year of birth

Year of birth	1999	2000	2001	2002	2003	2004	2005	2006	2007	2008
*Males*										

N	145	135	134	127	123	107	107	123	145	141

Mean	29.8	32.7	30.6	37.1	34.4	24.5	34.4	31.3	27	29.6

*k1*	*0.082*	*0.089*	*0.084*	*0.102*	*0.094*	*0.067*	*0.094*	*0.086*	*0.074*	*0.081*

*Females*										

N	114	113	88	87	99	106	112	108	105	107

Mean	39.6	35.0	35.8	43.1	29.6	23.8	26.3	31.9	31.8	31.0

*k1*	*0.109*	*0.095*	*0.098*	*0.118*	*0.081*	*0.065*	*0.072*	*0.087*	*0.087*	*0.085*

Breslow chi-square statistic (males vs. females):										

*P*-value	0.26	0.96	0.39	0.93	0.73	0.45	0.15	0.93	0.60	0.88

Mean survival time of the deceased in their year of birth (in days) and mean proportion lived during that year (*k1*), Flemish Region (Belgium), 1999-2008, by sex

### Mean time-to-event in the next year yet before the first anniversary

From Table 2 we learn that the mean survival time for those dying in the year after their year of birth yet before their first anniversary, expressed as a proportion of the calendar year *(k2)*, fluctuates between 0.43 (year of birth 2006) and 0.55 (2000) in males and between 0.36 (2002) and 0.67 (2008) in females. The average proportion for all observation years approaches 0.5, i.e. 0.502 in males and 0.495 in females.

**Table 2:** Mean survival time of the deceased in the year after the year of birth

Year of birth	1999	2000	2001	2002	2003	2004	2005	2006	2007	2008
*Males*										

N	24	28	34	26	15	21	27	23	13	23

Mean	171.8	200.8	190.4	170.1	186.3	190	185.1	157.3	193.4	188.7

*k2*	*0.470*	*0.549*	*0.522*	*0.466*	*0.510*	*0.520*	*0.507*	*0.431*	*0.529*	*0.516*

*Females*										

N	24	26	11	13	17	12	18	14	11	10

Mean	198.2	150.1	184.0	132.3	185.5	160.5	221.6	151.6	180.7	243.8

*k2*	*0.542*	*0.411*	*0.504*	*0.362*	*0.508*	*0.439*	*0.607*	*0.415*	*0.494*	*0.667*

Breslow chi-square statistic (males vs. females)										

*P*-value	0.44	0.042	0.74	0.23	0.96	0.40	0.34	0.60	0.89	0.15

Mean survival time of the deceased in the year after the year of birth yet before the first anniversary (in days) and mean proportion of a calendar year lived (*k2*), Flemish Region (Belgium), year of birth 1999-2008, by sex

Differences in survival graphs of the gender groups are generally not statistically significant on a yearly basis, the one exception being the year of birth 2000.

## Discussion

### Infant mortality rate

Infant mortality has reached very low levels. Our figures for the Flemish Region come close to 4.5‰ for boys and 3.5‰ for girls. Recent figures for Belgium show rates below 5‰ [5]. The low observed mortality levels testify to the important progress that has been made in this respect over the last century [6], particularly also in the more recent past [7].

Looking at Figure 3, however, it becomes clear that over the last decade the recorded level is stabilizing as if some bottom line were reached. For similar reasons, a threshold in the long term of 3‰ was applied in the 2008 federal population forecasts, referring to the then lowest level ever attained in a European country, i.e. Finland in 2002 [7]. Nevertheless, since the starting level was very low already, an overall decrease of about 20% over the last 10 years might still be labelled as relatively important. Moreover, it remains to be seen if a real threshold can be reached. Indeed, the infant death toll today largely consists of (extreme) preterm births that until recently would probably not have been considered as live births [8]. The definition of live births itself is changing within the high tech context of perinatal care. This implies that today's observations are only valid for the present situation.

### Mean time-to-event

The main concern for this paper was to find which values are valid for the mean survival time since birth. The survival time is expressed as a proportion of the calendar year, for either the infants dying in their year of birth (*k1*) or the infants dying in the following year but before their first anniversary (*k2*).

The average time-to-event in our database for the Flemish Region for infants who died in their year of birth (see base triangle a1 in Figure 1) was found to centre around 1 month (about 30 days), which results in a value for *k1 *equal to 0.08 or 0.09, regardless of gender. This matches with the mean survival time for that group of infants equal to 0.1 year as adopted by *Statistics Belgium*, the federal office for statistics in Belgium [9].

For the group of infants who died in the next year, yet before their first anniversary, the mean survival time since birth approximates 0.5 year (with an average value of 0.22 years in the time segment depicted by triangle a2' on the hypothetical cohort in Figure 1). Nevertheless, as the mortality rate here becomes very low, some broader random fluctuation in the mean survival time on a yearly basis surfaces.

In life tables with age at birth (model (b) in Figure 1), it is necessary to plug in some value for *k*, i.e. the mean proportion of the calendar year lived by infants deceased in their first year of life. This value can be seen as the weighted average of *k1 *and *k2*. So, for the group of male infants who died under age 1 in our study population (perceived to belong to one birth cohort), we could write: *k *= 0.1*(0.86) + 0.5*(0.14) = 0.156. From a practical point of view, *k *equal to 0.15 would do, which is the value adopted by *Statistic Belgium *[9].

There are however some caveats. First, there is no complete coverage of all births and deaths. Indeed, the Flemish registration system does not cover those births and deaths of residents of the Flemish Region that took place in the Walloon Region or abroad, and of which the birth or death certificates were not presented to the Flemish authorities. These are rare events and their absence should normally have no impact on our results. The missed infant deaths might be considered as non-identified right-censored cases (i.e. lost-to-follow-up). To give an idea of its rarity, its share in the total number of infant deaths was 2.1% (6 cases in 284 infant deaths under age 1 according to the National Register) for the calendar year 2007 (personal communication by Michel Willems of *Statistics Belgium*, 13/02/2012).

In a more theoretical sense, this problem of censored cases also pertains to infants that have migrated out of the region and possibly have died within the observation year. From migration data for the Flemish Region at our disposal, we learn that the age-specific emigration rate in the year of birth is small (e.g. 5‰ in 2007). Considering that about half of the infant deaths occur within the first week after birth, in which time period we do not expect families to migrate much, the impact of the censored cases must be very small indeed. Besides, in population statistics the deaths of persons who officially left the population are generally no longer taken into account.

Secondly, the calculation in days lived since birth tends to over-estimate the person-years lived by those who died in the very first days of life. If we were to count in hours instead, it would turn out that a) more infants died within the first period of twenty-four hours than recorded within the first calendar day (some on the second calendar day are then classified within the first natural day of life), and b) these infants lived much less than half a day on average. For instance, in the year 2007 we find in our study population that an extra 21% deaths have occurred on the first natural day of life compared to the first calendar day (resp. 86 vs. 71 infants). The mean survival time in hours is 3.5 hours, which makes for 0.15 days instead of the hypothesized 0.5 days of life. As roughly a quarter of the deaths among the infants who died in their year of birth occurred on the calendar day of birth itself, this may have some impact. When putting this to the test for infants that have died in the first three days of life in 2007, we find that the value for *k1 *starts to change on the 3-digit precision level (from 0.0795 to 0.0788). The test for 2008 gives similar results (*k1 *changes from 0.0827 to 0.0825). Obviously, such small changes are negligible in the context of determining life expectancy at birth.

### A need for harmonized statistics

Today, there is an important demand within the European Union for benchmarking, often with ranking of Member States (or their smaller regions) on some policy indicator. Generally, this is an interesting exercise provided equals are being compared with equals. For this reason, the European Demographic Observatory (ODE) of Eurostat published a manual with guidelines designed to harmonize algorithms for demographic indicators [10], including an appendix with a great deal of information on how to construct life tables [a reprint from [11]].

ODE stresses the fact that in the field of general population statistics both statistical quality and simplicity are key, which may call for a compromise. As births are usually followed up well in the year of birth, we think it feasible that at least for that time period (i.e. the base triangle on the Lexis diagram) a more precise assessment of the mean duration of life of deceased infants be taken into account. Here indeed, most of the infant deaths are concentrated, random variation is low and departure from a uniform distribution over time is largest. In our opinion, an estimate based on observations with a 1-digit precision may therefore meet the compromise.

### Gender differences

Female babies in our study population show a somewhat better risk profile than their male counterparts, although the differences are minimal and generally not statistically significant on a yearly basis. By the same token, our data do not support the need to specify different values for the *k-*factors according to gender.

This is not to say that other factors have no predictive value. For instance, a study on stillbirths and infant mortality among hospital births by mothers aged 25 years and over in 1999 in the Flemish Region has shown that the educational level of the mother is an important determinant of foetal and, to a lesser degree, early neo-natal infant mortality [12]. For certain social factors, it may be worthwhile performing a study to see to what extent this is reflected in the deceased infants' mean duration of life.

## Conclusion

Infant mortality in the Flemish Region of Belgium has reached very low levels. It remains to be seen, however, to what extent further progress will be possible.

As most of the deaths in the year of birth are concentrated within the first week after birth, *Statistics Belgium *imputes a value equal to 0.1 for the mean proportion of the calendar year lived by the deceased in the year of birth (*k1*) when calculating life expectancy. Our data fully support this value. We think such refinement on the 1-digit precision level is generally feasible in calculating life expectancy.

## Competing interests

The authors declare that they have no competing interests.

## Authors' contributions

EP planned the analysis and drafted the manuscript. HC was responsible for the dataset, took part in the analysis and revised the draft paper. EH was responsible for the data processing, took part in the analysis and commented on the draft paper. All authors read and approved the final manuscript.
